# Seizure control by decanoic acid through direct AMPA receptor inhibition

**DOI:** 10.1093/brain/awv325

**Published:** 2015-11-25

**Authors:** Pishan Chang, Katrin Augustin, Kim Boddum, Sophie Williams, Min Sun, John A. Terschak, Jörg D. Hardege, Philip E. Chen, Matthew C. Walker, Robin S. B. Williams

**Affiliations:** ^1^ 1 Centre for Biomedical Sciences, School of Biological Sciences, Royal Holloway University of London, Egham, TW20 0EX, UK; ^2^ 2 Department of Clinical and Experimental Epilepsy, Institute of Neurology, University College London, WC1N 3BG, UK; ^3^ 3 School of Biological, Biomedical and Environmental Sciences, University of Hull, Cottingham Road, Hull HU6 7RX, UK

**Keywords:** AMPA receptors, decanoic acid, epilepsy, ketogenic diet, seizure control

## Abstract

**See Rogawski (doi:10.1093/awv369) for a scientific commentary on this article.**

The medium chain triglyceride ketogenic diet is an established treatment for drug-resistant epilepsy that increases plasma levels of decanoic acid and ketones. Recently, decanoic acid has been shown to provide seizure control *in vivo*, yet its mechanism of action remains unclear. Here we show that decanoic acid, but not the ketones β-hydroxybutryate or acetone, shows antiseizure activity in two acute *ex vivo* rat hippocampal slice models of epileptiform activity. To search for a mechanism of decanoic acid, we show it has a strong inhibitory effect on excitatory, but not inhibitory, neurotransmission in hippocampal slices. Using heterologous expression of excitatory ionotropic glutamate receptor AMPA subunits in *Xenopus* oocytes, we show that this effect is through direct AMPA receptor inhibition, a target shared by a recently introduced epilepsy treatment perampanel. Decanoic acid acts as a non-competitive antagonist at therapeutically relevant concentrations, in a voltage- and subunit-dependent manner, and this is sufficient to explain its antiseizure effects. This inhibitory effect is likely to be caused by binding to sites on the M3 helix of the AMPA-GluA2 transmembrane domain; independent from the binding site of perampanel. Together our results indicate that the direct inhibition of excitatory neurotransmission by decanoic acid in the brain contributes to the anti-convulsant effect of the medium chain triglyceride ketogenic diet.

## Teaser

The MCT ketogenic diet, an established treatment for drug-resistant epilepsy, leads to an elevation of plasma decanoic acid and ketones. Chang *et al.* show that decanoic acid, rather than ketones, provides anti-seizure activity in several *ex vivo* rat models of epilepsy, likely through the direct inhibition of AMPA receptors.

## Graphical abstract

**Figure:**
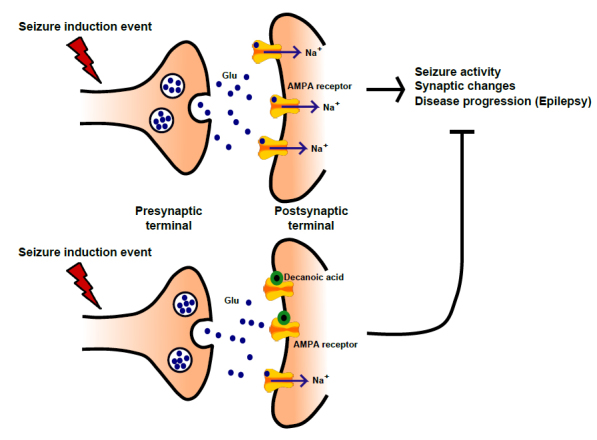
**See Rogawski (doi:10.1093/awv369) for a scientific commentary on this article.** The MCT ketogenic diet, an established treatment for drug-resistant epilepsy, leads to an elevation of plasma decanoic acid and ketones. Chang *et al.* show that decanoic acid, rather than ketones, provides anti-seizure activity in several *ex vivo* rat models of epilepsy, likely through the direct inhibition of AMPA receptors.

## Introduction

The medium chain triglyceride (MCT) ketogenic diet was first identified as a treatment for refractory epilepsy in 1971 (Huttenlocher *et al.*, 1971). It has provided one of the most effective therapeutic approaches for children with drug-resistant epilepsy (Liu, 2008; Neal and Cross, 2010; Levy *et al.*, 2012) and has recently been demonstrated to be effective in childhood epilepsy in a randomized control trial (Neal *et al.*, 2009). However, the diet has adverse gastro-intestinal related side effects, such as diarrhoea, vomiting, bloating, and cramps (Liu, 2008). Furthermore, it has also been shown that there is a high attrition rate for the diet, due to many patients finding the diet difficult to tolerate (Levy *et al.*, 2012). An understanding of the mechanism of action of the diet will not only provide insight into mechanisms underlying epilepsy, but also facilitate the development of novel drugs and better targeted dietary therapies that are effective in drug-resistant epilepsy, yet lack many of the adverse effects of the conventional MCT diet.

Understanding the therapeutic mechanism of the diet has been challenging (Rho and Sankar, 2008; Rho and Stafstrom, 2011). It was initially assumed that ketone production was the key to the diet’s antiepileptic effect, but there is a poor correlation between serum ketones and seizure control (Likhodii *et al.*, 2000; Thavendiranathan *et al.*, 2000). Moreover, *in vitro* and *in vivo* studies of the anticonvulsant effects of ketones have proved inconclusive (Likhodii *et al.*, 2000; Thavendiranathan *et al.*, 2000). In addition to ketones, the diet also causes an increase in plasma levels of the two fatty acids provided in MCT oil, the straight chain, 10-carbon decanoic acid, and the eight-carbon octanoic acid (Haidukewych *et al.*, 1982; Sills *et al.*, 1986*a*). Recently, it has been established that decanoic acid has antiseizure effects at clinically relevant concentrations *in vitro* and *in vivo* (Chang *et al.*, 2013; Wlaz *et al.*, 2015) but octanoic acid does not, and with previous *in vivo* pharmacokinetic data indicating that decanoic acid penetrates the blood–brain barrier (Oldendorf, 1973), these data suggest that decanoic acid directly contributes to the therapeutic effect of the MCT ketogenic diet. Indeed, *in vitro*, decanoic acid is more potent than valproic acid [a branched chain fatty acid isomer of octanoic acid, that is commonly used in the treatment of epilepsy (Chang *et al.*, 2013), and which has been shown to act on phosphoinositide signalling in seizure control (Xu *et al.*, 2007; Chang *et al.*, 2012, 2014*a*)]. Thus, it is unclear if ketones or decanoic acid provide direct, acute antiseizure effects during administration of an MCT diet, and by what mechanism. These questions are addressed here.

## Materials and methods

### Animals

Male Sprague-Dawley rats were housed in cages under controlled environmental conditions (24–25°C; 50–60% humidity; 12-h light/dark cycle) with free access to food and water. All efforts were made to minimize the number of animals used. All experiments were performed under personal and project licenses approved by the Home Office, London, UK under regulations of the UK Animal (Scientific Procedures) Act, 1986.

### Slice preparation

Male Sprague-Dawley rats (postnatal Days 19–30) were sacrificed using an overdose of isoflurane or pentobarbitone (500 mg/kg). After decapitation the brain was rapidly removed, the hippocampus or entorhinal cortex-hippocampus slices was dissected from the brain and 350-µM thick transverse slices were prepared on a VT1200S vibratome (Leica) or Vibratome (Vibratome® 1500 sectioning system, Intracell). The slicing was performed in ice-cold sucrose-based solution containing (in mM): 75 sucrose, 87 NaCl, 22 glucose, 2.5 KCl, 7 MgCl_2_, 1.25 NaH_2_PO_4_, 0.5 CaCl_2_ and 25 NaHCO_3_, pH 7.4, 315–330 mOsm equilibrated with 95% O_2_ plus 5% CO_2_. The slices were then stored in a continuously oxygenated humidified interface holding chamber containing oxygenated artificial CSF (in mM): 120 NaCl, 22 glucose, 2.5 KCl, 1.3 MgSO_4_, 1 NaH_2_PO_4_, 25 NaHCO_3_ and 2.5 CaCl_2_, pH 7.4 296 mOsm, where they recovered for at least 1 h before use.

### Electrophysiology

#### *In vitro* seizure model

During the experiment, slices were transferred from the interface chamber into a submerged recording chamber, designed to optimize wash-in and wash-out of drugs, and continuously perfused using gravity feed at 3–6 ml/min with prewarmed (36°C) oxygenated artificial CSF (95% O_2_, 5% CO_2_). Field potentials were recorded with a glass microelectrode (1–2 MΩ) filled with artificial CSF solution placed in stratum radiatum of CA1 and were filtered at 1 kHz and digitized at 2 kHz (using an npi EXT-02F extracellular amplifier recorded with WinEDR software). In the pentylenetetrazol model, the epileptiform (paroxysmal) activity was induced by application of pentylenetetrazol (2 mM) to the perfusate and [K^+^] was increased (to 6 mM); in low-Mg^2+^ model, the epileptiform activity was elicited by using Mg^2+^-free artificial CSF. Once the frequency of the paroxysmal activity was stable for at least 10 min, compounds were applied to the perfusate for the following 40 min, and washed out for a remaining 20 min. The anticonvulsant effects were evaluated by measuring the change in the frequency of the discharges at minute intervals. The discharge frequency was then averaged every 5 min during the experiment and normalized to baseline. The compounds applied in this study included: 1% dimethyl sulphoxide (DMSO), acetone (10 mM, Sigma), (±)-sodium 3-hydroxybutyrate (BHB) (10 mM, Sigma), and decanoic acid (1 mM, Alfa Aesar Pty). Acetone and decanoic acids were prepared as 1000× stocks in DMSO, and BHB was prepared as 100× stock. Stocks were dissolved in artificial CSF to achieve their final concentrations during experiments, and where applicable, experiments included constant levels of DMSO.

#### Whole-cell patch clamp

For electrophysiological recording, the slices were placed in a recording chamber constantly perfused with 32–34°C oxygenated artificial CSF solution using a gravity-driven perfusion system. Whole-cell patch clamp recordings were performed on CA1 pyramidal cells (input resistance 330 ± 70 MΩ) visualized using an infrared differential contrast imaging system. For patching, standard walled borosilicate glass pipettes with a resistance of 2.5–3.5 MΩ were used, filled with an intracellular pipette solution containing (in mM): 120 Cs-methanesulphonate, 10 HEPES, 0.2 EGTA, 8 NaCl, 0.2 MgCl_2_, 2 Mg-ATP, 0.3Na-GTP, 5 QX314-Br, 10 phosphocreatine, pH adjusted to 7.2 and osmolality adjusted to 296 mOsm. Series resistance was monitored throughout experiments using a −5 mV step command and cells showing a >20% change in series resistance, a series of resistance of >20 MΩ or an unstable holding current were rejected. Recordings of excitatory postsynaptic currents (EPSCs) were performed in the presence of DL-APV (Dl-2-amino-5-phosphonopentanoic; 100 µM), picrotoxin (100 µM) and CGP55845 (5 µM) to block NMDA-, GABA_A_- and GABA_B_-receptors, respectively. Decanoic acid (Sigma) was added to the perfusion solution. EPSCs were evoked by stimulation of Schaffer collaterals. Paired pulses were evoked with stimulations separated by 50 ms. Inhibitory postsynaptic current (IPSCs) were recorded in the presence of DL-APV (100 µM), NBQX (10 µM) and CGP55845 (5 µM) to block NMDA-, AMPA- and GABA_B_-receptors, respectively.

Recordings were obtained using a MultiClamp 700B amplifier (Axon instruments) and filtered at 4 kHz, digitized at 10 kHz, and stored on a PC. LabView8 (National instruments) was used for data acquisition and off-line analysis.

### *In vitro* RNA transcription of AMPA receptor subunits

The AMPA receptor (flip isoform) cDNAs inserted in a SP6 polymerase expression vector (pSP6T) were a generous gift from Prof. Ralf Schoepfer (NPP, UCL). RNA was transcribed *in vitro* from MluI linearized transcripts using the SP6 Promega RiboMAX® RNA synthesis kit according to manufacturer’s protocols except for the addition of 0.75 mM capping nucleotide m^7^G(5’)ppp(5’)G (Promega) and 1.6 mM GTP. cRNA concentrations and integrity were estimated by the intensity of fluorescence bands in RNA denaturating gels. AMPA receptor cRNAs were mixed in a nominal 1:1 ratio and ∼5 ng was injected per oocyte.

### Oocyte preparation and injection

*Xenopus laevis* oocytes were purchased from the European *Xenopus* Resource Centre, University of Portsmouth. Stage V to VI oocytes were mechanically dissected and then subjected to gentle shaking for ∼30–50 min at room temperature with modified Barth’s solution (in mM): 88 NaCl, 1 KCl, 2.4 NaHCO_3_, 0.82 MgCl_2_, 0.77 CaCl_2_, 15 Tris-Cl, adjusted to pH 7.4 with NaOH (Sigma-Aldrich), supplemented with 50 IU/ml penicillin and 50 µg/ml streptomycin (Invitrogen) and 50 µg/ml tetracycline (Sigma-Aldrich) and 1% collagenase (type 1A). Healthy oocytes were manually defolliculated and the injections of cRNA for homomeric subunits alone (GluA1), or heteromeric mixtures of two subunits together (GluA1/GluA2 or GluA2/GluA3) were made using an automated Drummond Nanoinject II injector. The oocytes were then incubated at 17°C in modified Barth’s solution for at least 48 h before use in electrophysiological recordings.

### Two-electrode voltage clamp recordings from oocytes

Experiments were performed at room temperature (∼21–23°C). An oocyte was placed in a recording chamber (0.3–0.5 ml volume) and perfused with ND96 solution (96 mM NaCl, 2 mM KCl, 1.8 mM CaCI_2_, 1 mM MgCl_2_, 5 mM HEPES, with pH adjusted to 7.5). Current and voltage electrodes were filled with 300 mM KCl and made from thin-walled borosilicate glass (GC150TF-7.5, Harvard Apparatus) using a PC-10 electrode puller (Narashige Instruments) and had resistances of 0.5–2 MΩ. Oocytes were voltage-clamped to a holding potential of −50 mV or −60 mV using a Turbo TEC-03 amplifier (npi electronics). Compounds were dissolved in distilled water or DMSO and dissolved in bathing solution to achieve their final concentrations during experiments, and were applied under gravity flow during the experiment by using a multi-valve perfusion system (VC3-8C, ALA Scientific Instruments). The bath solutions were perfused at a rate of 10 ml/min. Recordings were filtered at 20 Hz and digitized at 100 Hz (Digidata 1322A, Molecular Devices) before recording to computer hard disk. Data acquisition was performed using the Windows PC-based program, WinEDR v3.0.6 (John Dempster, University of Strathclyde, UK).

### Molecular dynamics studies

SMILES format of each ligand was converted to 3D PDB files using Open Babel (Ver. 2.3.2). The obconformer function was then used to find the lowest energy conformation from 5000 test conformers after 100 geometry optimization steps. A GluA2 receptor molecule (Protein Data Bank Code 3KG2) downloaded from the Research Collaboratory for Structural Bioinformatics Protein Data Bank (RSCB PDB) was processed using AutoDock (Ver 1.5.6) in which solvent water molecules, duplicate subunits (B, C, and D), and bound ligands (zinc, β1.5.6) in which solvent water molecules, ΖΚ1 [7-morpholin-4-yl-2,3-dioxo-6-(trifluoromethyl)-3,4-dihydroquinoxalin-1(2H)-yl]methyl}phosphonic acid)] were removed. Polar hydrogens were retained after the addition of Kollman charges and the distribution of charge deficit. Beginning with the ligand in its lowest energy conformation, each was then allowed to rotate freely about its single bonds during the docking process. For 3KG2, the docking grid was set in a 126-unit box centred at *x*: 16.971, *y*: 40.698, and *z:* −106.466 with 0.528 Å spacing; this encompassed the entire transmembrane domain of the A subunit (Supplementary Fig. 6). The docking search parameters used AutoDock’s Genetic Algorithm with 25 runs of 2.5 million evaluations and an initial population size of 250. Upon completion of docking of each ligand, the numbers of conformations as well as the range of their binding energies were noted for each cluster of docking locations. The amino acid residues within 6 Å of the lowest energy conformation of each bound ligand were noted. The residues most frequently participating in binding with the various ligands were targeted as the most likely common binding site for 3KG2 (Supplementary Fig. 3).

### Statistical analysis

The data from the electrophysiology experiments were analysed using GraphPad Prism software and SPSS (IBM) with IC50 values calculated using *y* = 100 / (1 + 10^(^*^x^*^ − logIC50)^). Statistical analysis was performed using ANOVA with Tukey *post hoc* test, paired *t*-test or unpaired *t*-test.

## Results

### Decanoic acid, not ketones, acutely inhibits epileptiform activity

**Figure 1:**
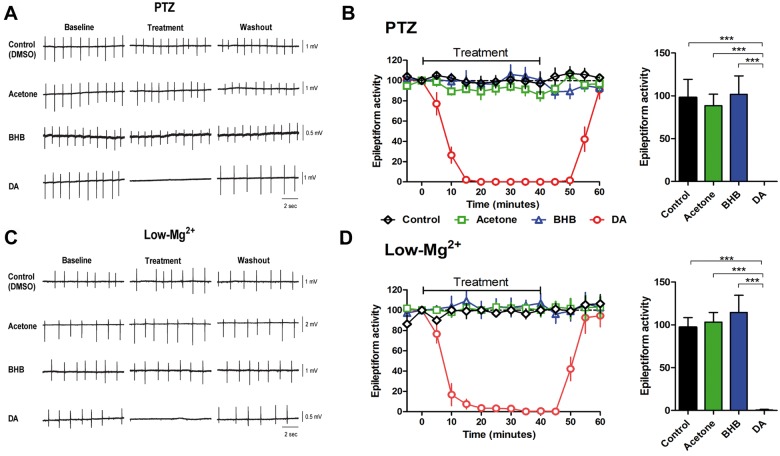
**Decanoic acid but not ketones acutely reduce epileptiform activity in two acute *ex vivo* models.** Epileptiform activity was monitored using two *ex vivo* rat hippocampal slice models, following treatment with decanoic acid (DA, 1 mM) or ketones acetone and β-hydroxybutyrate (BHB) (both at 10 mM) or with solvent-only (Control). (**A**) Example trace recording of epileptiform activity (burst discharges) in hippocampal slices induced by application of pentylenetetrazol (PTZ, 2 mM, K^+^ 6 mM), a model for generalized seizures, and following treatment, where (**B**) the frequency of epileptiform activity is plotted against time, following treatment, and also shown as a comparison of the mean (±SD) frequency of burst discharges averaged from 20 to 40 min post-compound addition. (**C**) Example trace recording of epileptiform activity (burst discharges) in hippocampal slices induced by low-Mg^2+^ conditions, as a model for drug-resistant seizures, and following treatment, where (**D**) the frequency of epileptiform activity is plotted against time, and also shown as a comparison of the mean (±SD) frequency of burst discharges averaged from 20 to 40 min post-compound addition. Significance indicated by ***P < 0.001 compared to control (ANOVA with Tukey *post hoc* test). All data were normalized to baseline. Data are provided from between *n = *4 and 7 repeats.

We initially assessed whether the acute antiseizure effects of the MCT ketogenic diet are related to a direct effect of decanoic acid or ketone bodies (β-hydroxybutyrate and acetone). Here we used two different *in vitro* models of epileptiform activity generated by decreasing GABAergic inhibition (pentylenetetrazol) (Fig. 1A–C) or potentiating NMDA receptor currents (low magnesium) (Fig. 1D–F). In both models, even at high concentrations (10 mM), ketone bodies did not have any effect on epileptiform activity. In contrast, at a concentration at which valproic acid, a branched chain fatty acid and long-established antiepileptic drug, only modifies seizure activity in these models (1 mM) (Chang *et al.*, 2012, 2013), decanoic acid completely blocked epileptiform activity in both models (Fig. 1). This supports an antiseizure effect of the decanoic acid component of the MCT diet, rather than diet-derived ketones.

### Decanoic acid acutely inhibits excitatory postsynaptic currents

**Figure 2:**
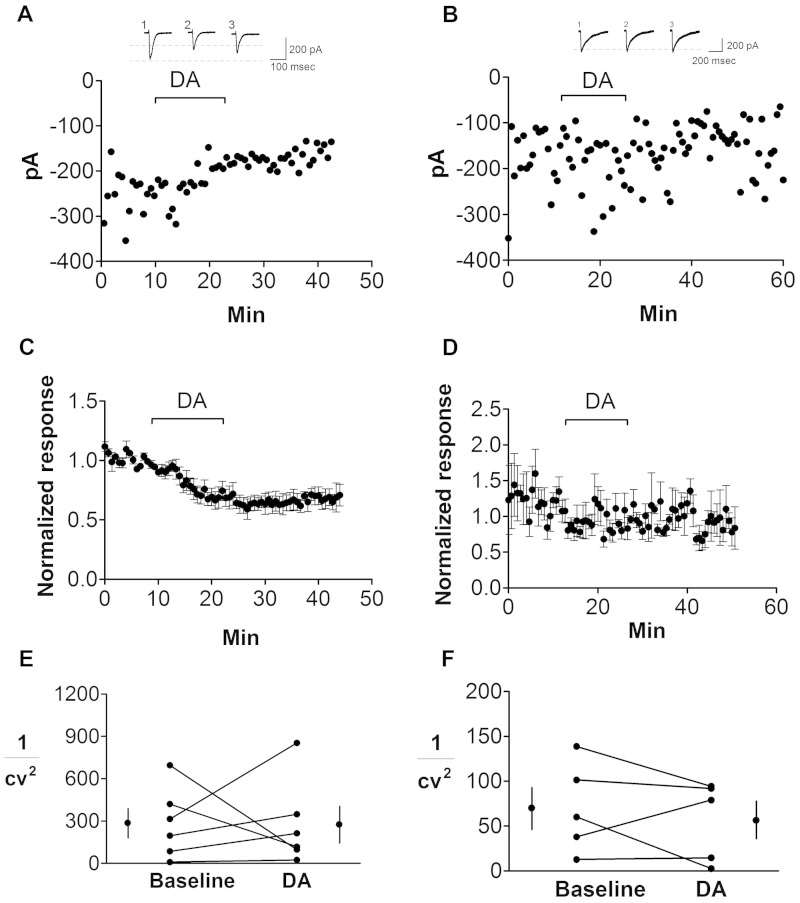
**The effect of decanoic acid on EPSCs and IPSCs.** Currents were recorded from hippocampal CA1 pyramidal cells following exposure to decanoic acid (DA; 300 μM). (**A**) Representative electrophysiological recordings show reduced evoked EPSC amplitude following application of decanoic acid. The insert provides the average of 10 traces from the same cell indicating the shape of single EPSCs before (1), during (2) and after (3) application of decanoic acid. (**B**) Representative electrophysiological recordings show no effect of decanoic acid on IPSCs. (**C**) Summary data showing the effect of decanoic acid on mean normalized EPSCs ± SEM (*n = *6). (**D**) In contrast, decanoic acid had no effect on mean normalized IPSCs ± SEM (*n = *6). Decanoic acid did not change 1/CV^2^ (CV, coefficient of variation) of EPSC (**E**) or IPSC (**F**) amplitudes with means ± SEM pre- and post-decanoic acid treatment as indicated, consistent with a post-synaptic locus of action.

We next asked what the molecular mechanisms for the effect of decanoic acid in seizure control are likely to be. As most antiepileptic drugs modify synaptic transmission or neuronal excitability, we first tested the effect of decanoic acid on either excitatory or inhibitory neurotransmission by determining the effect of decanoic acid on evoked EPSCs and IPSCs in acute *ex vivo* hippocampal slices. For these experiments, we performed whole-cell patch clamp recordings from CA1 pyramidal neurons and stimulated the stratum radiatum in the presence of NMDA-, GABA_A_- and GABA_B_-receptor blockers to isolate AMPA receptor mediated EPSCs or in the presence of NMDA-, AMPA- and GABA_B_-receptor blockers to isolate GABA_A_ receptor mediated IPSCs and applied 300 µM (52 µg/ml) decanoic acid [similar to peak serum concentration of children on the MCT diet (Sills *et al.*, 1986*a*), and to the serum and brain concentrations necessary for acute seizure effects in mice (Wlaz *et al.*, 2015)]. Decanoic acid decreased the EPSC amplitude by 38.9 ± 5.5%, (*n = *6; *P < *0.001; Fig. 2A and C), but had no significant effect on evoked IPSCs (Fig. 2B and D). Furthermore, decanoic acid (300 μM) had no effect on 1/CV^2^ (CV, coefficient of variation) for EPSCs (decanoic acid versus baseline; *n = *6: *P = *0.95; Fig. 2E) or IPSCs (decanoic acid versus baseline; *n = *6: *P = *0.95; Fig. 2F), and did not affect the paired pulse ratio for EPSCs (decanoic acid versus baseline; *n = *6; *P = *0.69 Supplementary Fig. 1A and B), consistent with a postsynaptic locus of action. At a concentration comparable to the steady state of decanoic acid in children on the MCT diet (100 µM) (Sills *et al.*, 1986*a*); decanoic acid still significantly reduced EPSC amplitudes by 17.0 ± 5.6% (*P = *0.01; *n = *6). Our results are, therefore, consistent with a significant (direct or indirect) effect of decanoic acid on postsynaptic excitatory (AMPA) receptor activity.

### Decanoic acid directly inhibits AMPA receptor activity

**Figure 3:**
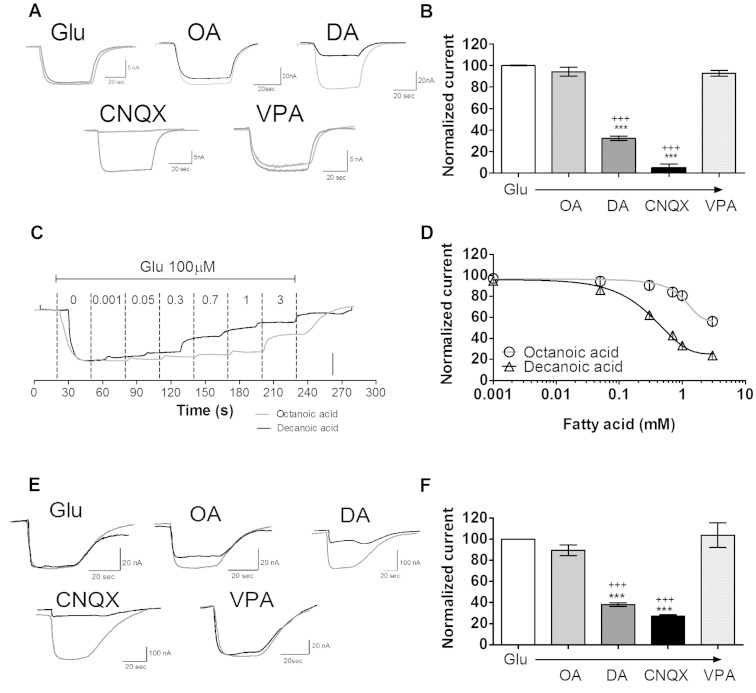
**The direct effect of decanoic acid on AMPA receptor-mediated currents.** In these experiments, *Xenopus* oocytes were used to express AMPA (GluA2/A3 or GluA1) receptors, and currents were measured following application of L-glutamate (100 µM) with membrane potential clamped to −50 mV. (**A**) Representative trace recordings for GluA2/3, showing the effect of medium chain fatty acids decanoic acid (DA), octanoic acid (OA) and VPA (all at 1 mM) and the AMPA receptor inhibitor CNQX (30 μM) on inward currents; and (**B**) summary of mean normalized currents (±SEM). (**C**) Representative current traces of inhibitory dose-response curves for octanoic or decanoic acid. (**D**) Mean inhibitory dose-response curves for octanoic or decanoic acid, graphs show means ± SEM. (**E**) Representative trace recordings for GluA1, showing the effect of medium chain fatty acids and VPA on inward currents. (**F**) Summary of mean normalized currents (±SEM). Statistical analysis was performed using ANOVA with Dunnett’s *post hoc* test. *P < 0.05, ***P < 0.001, compared to control; ^+++^*P < *0.001, compared to solvent only (DMSO). Scale bars = 15 nA for octanoic acid and 5 nA for decanoic acid.

To determine whether decanoic acid has a direct effect on AMPA receptor activity, we employed a heterologous expression system in which AMPA receptor subunits (GluA2/3) were expressed in *Xenopus* oocytes and agonist elicited inward currents were used to test the inhibitory activity of decanoic acid. In these experiments, we measured the effect of decanoic acid, octanoic acid and valproic acid (all at 1 mM; *n = *12), on currents elicited by application of 100 µM L-glutamate. In line with its anticonvulsant efficacy (Chang *et al.*, 2012, 2013), decanoic acid markedly reduced AMPA receptor currents (32.4 ± 2.0% of control, *n = *24, *P < *0.001; Fig. 3A and B), whereas octanoic acid had no effect. Valproic acid also showed no action on AMPA receptor currents. Inhibitory dose-response curves showed that the effect of decanoic acid was dose dependent (Fig. 3C and D), and more potent (mean IC50 = 0.52 ± 0.02 mM, *n = *12) than that of octanoic acid (mean IC50 = 3.82 ± 0.03 mM, *n = *10). The inhibition of glutamate elicited inward currents by decanoic acid was also seen in oocytes expressing GluA1 homomeric channels (Fig. 3E and F). Decanoic acid also inhibited AMPA receptors following activation with the selective high-affinity AMPA receptor agonist, AMPA (30 µM; 16.7 ± 2.7% of AMPA alone; *n = *8; *P < *0.001) (Supplementary Fig. 2). This inhibitory effect indicates a direct, structurally specific inhibition of AMPA receptor currents by decanoic acid.

**Figure 4:**
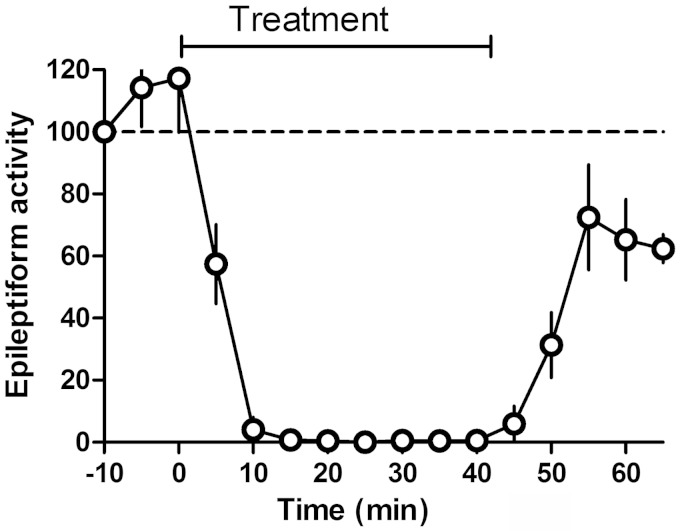
**The effect of AMPA receptor antagonism with GYKI 52466 on epileptiform activity.** Epileptiform activity induced by application of pentylenetetrazol (2 mM, K^+^ 6 mM) was monitored following treatment with the selective AMPA receptor antagonist GYKI 52466 at a concentration (50 µM) that results in approximately the same (∼60–70%) decrease in AMPA receptor responses (± SD) as is observed with 1 mM decanoic acid (*n* = 4).

We next investigated if AMPA receptor inhibition could block epileptiform activity *in vitro* using the hippocampal/pentylenetetrazol seizure model. Here we tested whether GYKI 52466—a non-competitive AMPA receptor antagonist—at a concentration that gives the same degree of AMPA receptor antagonism as 1 mM decanoic acid (Arai, 2001). GYKI 52466 (50 µM) completely blocked *in vitro* epileptiform activity induced by pentylenetetrazol (Fig. 4), indicating that the AMPA receptor antagonism of decanoic acid is sufficient to explain its pharmacological effect *in vitro*.

**Figure 5:**
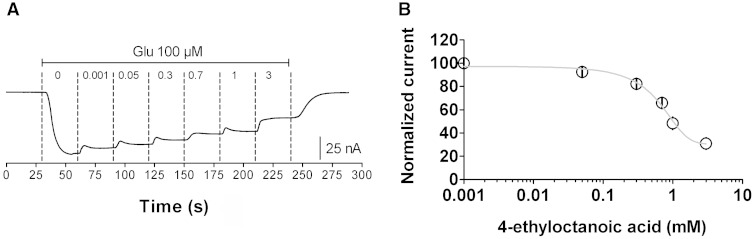
**The effect of 4-ethyloctanoic acid on AMPA (GluA2/A3) mediated current in *Xenopus* oocytes induced by L-glutamate**. (**A**) Representative trace recording showing the effect of 4-ethyloctanoic acid at indicated concentrations on AMPA (GluA2/A3) current following application of glutamate (100 µM). (**B**) Inhibitory dose-response curves of 4-ethyloctanoic acid (*n* = 4) in the presence of glutamate (100 μM), with data present as means ± SEM. The responses are normalized to the maximal current response induced by application of L-glutamate for each recording.

We have previously observed that chemical modification of medium chain fatty acids can increase their potency against multiple *in vitro* and *in vivo* seizure models (Chang *et al.*, 2012, 2013, 2014*a*). For example, although octanoic acid shows little seizure control in these models, a branched derivative, 4-methyloctanoic acid is strongly active. We therefore examined the potency of 4-methyloctanoic acid for inhibitory activity at AMPA receptors (GluA2/3) (Fig. 5). This compound showed strong inhibitory activity [IC50 0.84 ± 0.04 mM (*n = *14)], consistent with that shown for seizure control. These data are consistent with medium chain fatty acid derivatives providing direct AMPA receptor inhibition relating to seizure control.

### Decanoic acid inhibition of AMPA receptor activity is subunit dependent, non-competitive and voltage dependent

**Figure 6:**
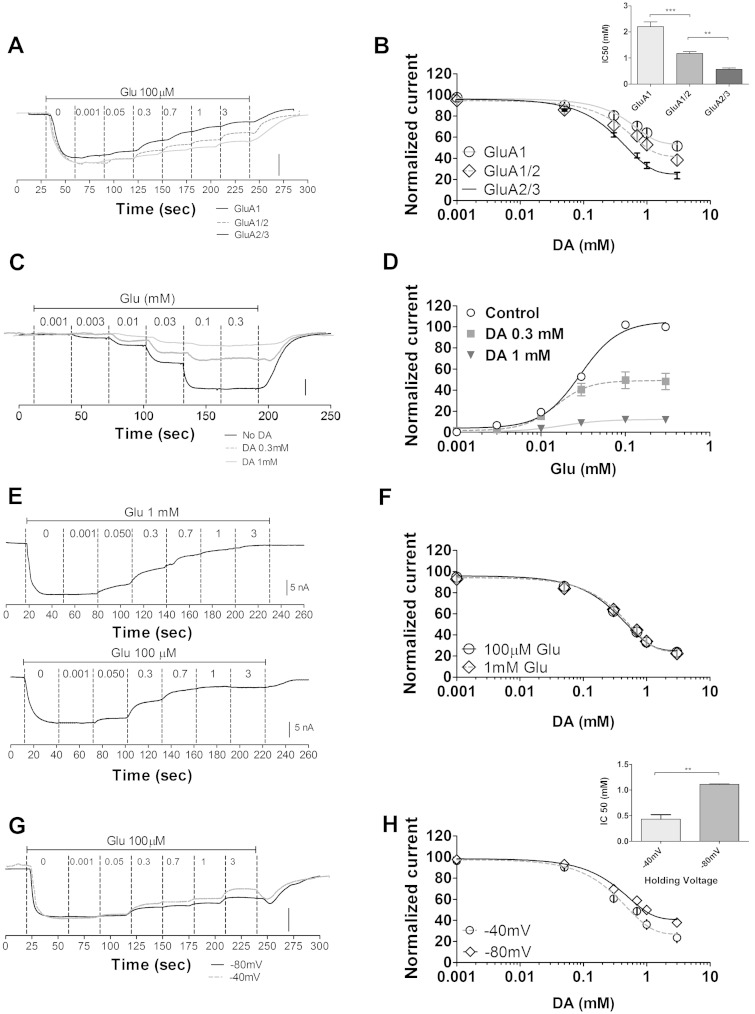
**Characterization of decanoic acid-dependent AMPA receptor inhibition**. In these experiments, *Xenopus* oocytes were used to express various AMPA receptor combinations, and glutamate-elicited currents were measured in the presence of varying decanoic acid concentrations. (**A**) Representative current trace showing different AMPA receptor subunit combinations (GluA1; GluA1/2; GluA2/3) following exposure to varying decanoic acid concentrations (0.001, 0.05, 0.3, 0.7, 1 and 3 mM) on current following application of glutamate (at 100 µM). Scale bars = 200 nA for GluA1 and GluA1/2; 20 nA for GluA2/3, and (**B**) mean inhibitory dose-response curves of decanoic acid from different AMPA receptor combinations (*inset* provides IC50 values) (*n = *12). (**C**) Representative traces from electrophysiological recordings showing the effect of glutamate (0.001, 0.003, 0.01, 0.03, 0.1, 0.3 mM) on GluA2/3 currents in the presence of decanoic acid (at 0.3 mM or 1 mM). Scale bar = 5nA (no decanoic acid); 2 nA (0.3 mM decanoic acid); and 4 nA (1.0 mM decanoic acid). (**D**) Quantitative evaluation of decanoic acid potency against GluA2/3 currents showing mean glutamate concentration-response curves (*n = *5 for each treatment) where responses are normalized to the maximal current for each recording in the absence of decanoic acid. (**E**) Representative current trace of the inhibitory dose–response curves for decanoic acid in the presence of glutamate (100 µM; *n = *20 and 1 mM; *n = *6) for GluA2/3 receptors and (**F**) mean inhibitory dose–response curves for decanoic acid against GluA2/3 in the presence of 100 µM or 1 mM glutamate show little change, suggesting a non-competitive inhibition of AMPA mediated current with respect to glutamate. (**G**) Representative current traces of the voltage dependence of decanoic acid inhibitory activity, where inhibition curves for decanoic acid in the present of glutamate (100 µM) for GluA2/3 receptors with voltage clamped to −80 and −40 mV. Scale bars = 200 nA and 20 nA, respectively. (**H**) Mean inhibitory dose-response curves at −80 and −40 mV (*inset* provides IC50 values) (*n = *6).

We next explored the subunit-dependency of decanoic acid inhibition on AMPA receptors. We employed oocytes expressing a homomeric (GluA1 subunit), and heteromeric (GluA1/2 and GluA2/3) AMPA receptors and measured currents following glutamate treatment (100 μM). Similar to the previous experiments, decanoic acid had a potent inhibitory effect on homomeric GluA1 AMPA receptors (Fig. 6A and B; IC50 = 2.09 mM; *P < *0.001 compared to solvent only), whereas octanoic acid and valproic acid had no effect. Decanoic acid’s potency was greater at the GluA1/2 heteromeric complex (Fig. 6A and B; IC50 = 1.16 mM; *P < *0.001 compared to GluA1 homomer), and even greater at the GluA2/3 heteromeric complex (Fig. 6A and B; IC50 = 0.52 mM; *P < *0.001 compared to GluA1/2 heteromer). This suggests that decanoic acid is a broad spectrum AMPA receptor inhibitor, but with differing potency at specific subunit combinations.

We further investigated the nature of decanoic acid-dependent AMPA receptor inhibition. We measured glutamate dose-response curves in the absence and presence of either 0.3 mM or 1 mM decanoic acid using GluA2/3 (Fig. 6C and D). Competitive inhibition would be predicted to shift the dose-response curves to the right (increasing the EC50 but with the same maximal response). However, in contrast, we observed a significant reduction in mean EC50s for glutamate in the presence of decanoic acid compared to control [from 0.029 mM for control to 0.015 mM and 0.018 mM for 0.3 mM (*P < *0.001) and 1.0 mM (*P* < 0.01) decanoic acid, respectively; Fig. 6D] and also a marked reduction in the maximal response at increasing decanoic acid concentrations. Such observations indicate that decanoic acid displays non-competitive inhibition at AMPA receptors. Furthermore, we also compared the mean IC50s of decanoic acid in the presence of low (100 μM) and high (1 mM) glutamate concentrations (Fig. 6E and F). Application of decanoic acid resulted in a concentration-dependent reduction in the glutamate-induced (100 µM) AMPA receptor current with an IC50 value of 0.52 ± 0.02 mM (*n = *12; Fig. 6E and F). Repeating these experiments at higher glutamate concentrations (1 mM) did not alter the inhibitory effect (0.54 ± 0.03 mM, *n = *10; Fig. 6E and F), suggesting that decanoic acid inhibition is not reversed by higher glutamate concentrations and is acting as a non-competitive inhibitor of AMPA receptors. This indicates that decanoic acid is likely to bind to a different region of AMPA receptors from that to which glutamate binds, and that this inhibitory mechanism may be shared amongst a number of fatty acids with anti-seizure activity.

We then investigated the voltage-dependence of direct AMPA receptor inhibition by decanoic acid using GluA2/3. Here, we clamped the voltage across the oocyte membrane at different membrane potentials, and examined changes in inhibitory activity of decanoic acid on AMPA-dependent current that may occur during synaptic depolarization. In these experiments, decanoic acid shows enhanced AMPA inhibition at the more depolarized membrane potential, −40 mV, where the IC50 is 0.43 ± 0.09 mM (*n = *6) in comparison to −80 mV, where the IC50 is 1.11 ± 0.05 mM (*n = *6) (Fig. 6G and H).

### Decanoic acid binds in the channel of AMPA receptors

**Figure 7:**
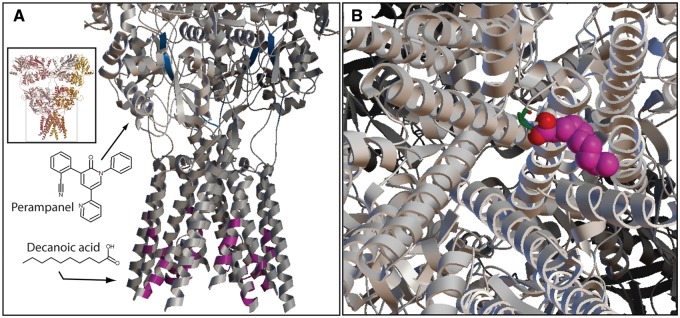
**Decanoic acid binding of AMPA receptors.** A molecular modelling approach was taken to investigate binding sites of decanoic acid on AMPA receptors using the active protein configuration (3KG2) of GluA2 and modelling residues within 6 Å of the ligand. (**A**) Decanoic acid is predicted to bind within the channel region of the receptor (magenta) on the M3 helices (overall structure shown in *inset*). Using this modelling approach, the known AMPA receptor agonist, perampanel, is predicted to bind to the linker region between the S1 glutamate binding domain and the channel pore (blue). (**B**) View from the intracellular side down the axis of the ion channel pore. Space-filled molecule is decanoic acid (magenta carbons and two red carboxylic acid oxygen molecules) binding at Pro584 (green) of one subunit with the equivalent binding of decanoic acid at the other three subunits omitted for clarity.

We next sought to identify a potential binding site for decanoic acid within the AMPA receptor using a modelling approach with the AMPA-sensitive, homotetrameric, rat GluA2 receptor (3KG2). *In silico* analysis was used to evaluate the binding of both decanoic acid and perampanel in the transmembrane domain of 3KG2 (Morris *et al.*, 2009), where over 2.5 million confirmations of decanoic acid and 3KG2 were evaluated, and the 25 conformations producing the lowest binding energy were used to define specific binding locations within the receptor. From these, the frequency of individual amino acid residues participating in binding (i.e. within 6 Å of decanoic acid) were determined (Supplementary Fig. 3). The most frequent residues involved in binding decanoic acid span residues 584–590 (Fig. 7A and B) (Supplementary Fig. 3). This coincides with the M3 helix of the transmembrane domain and, specifically, those residues found to influence the inward current through the receptor and thought to be involved in gating. We also examined the site for perampanel, a recently introduced treatment for partial onset seizures and showing efficacy in drug resistant epilepsy (Russo *et al.*, 2012; Rektor, 2013). Our modelling approach predicted perampanel to bind to a region in the border between S1 and the channel, consistent with that identified in other reports (Szenasi *et al.*, 2008; Rogawski, 2011). These data suggest that decanoic acid binds directly to the AMPA receptor channel, at a location separate from that of perampanel, and thus may exhibit a different physiological effect to perampanel.

## Discussion

The predominant mechanism of ketogenic diets, as the name suggests, has been widely considered to be through the production of ketone bodies (Bough and Rho, 2007; Rho and Stafstrom, 2011), such as 3-hydroxybutyrate and acetoacetate, since these are found to be elevated in the plasma of patients on the diet (Huttenlocher, 1976). However, a correlation between blood ketone levels and seizure control in patients and animal models has not been consistently found (Thavendiranathan *et al.*, 2000). Our study suggests that decanoic acid, rather than ketones, may provide the direct molecular mechanism of MCT ketogenic diet, in two *ex vivo* models for drug-resistant epilepsy, although ketones are likely to provide other benefits (Bough and Rho, 2007; Rho and Sankar 2008; Rho and Stafstrom, 2011; Kim *et al.*, 2015). This is further supported by other studies showing that ketone bodies do not directly alter excitatory or inhibitory hippocampal synaptic transmission (Thio *et al.*, 2000) nor affect epileptiform activity (either interictal or ictal activity) induced by 4-aminopyridine in hippocampal-entorhinal cortex slices (Thio *et al.*, 2000), although ketones have been shown to modulate vesicular glutamate transporter activity at presynaptic sites (Juge *et al.*, 2010). Nevertheless, there may be circumstances, when ketones do play a greater role such as in Glut1 deficiency, in which ketones from the ketogenic diet provide the brain with an alternative energy source (Veggiotti and De Giorgis, 2014). Moreover, the ketogenic diet in the longer term may modify metabolic and gene expression, which could have important disease-modifying effects (Masino *et al.*, 2011; Kim *et al.*, 2015). Also, the ketogenic diet (with reduced glucose) decreases cellular pyruvate/oxaloacetate concentrations, which has recently been shown to hyperpolarize neurons through an effect on neuronal potassium currents (Sada *et al.*, 2015).

The classical (long chain triglyceride; LCT) ketogenic diet, which has been shown to be as effective as the MCT diet (Neal *et al.*, 2009), provides up to 90% of the calories in the diet as fat and is extremely restrictive. In contrast, the MCT diet allows less energy to be provided as total fat (typically resulting in a total fat intake of 65–75% energy) allowing for a less restrictive and more varied diet. The implication of our results for the mechanism of action for the classical diet are yet to be explored; however, long chain fats are metabolized to shorter chain fats and it would thus be expected that serum medium chain fats will be increased. Future studies to monitor brain medium fatty acid levels in animal models and in patient plasma during ketogenic diets will be necessary to determine the role of fatty acids in the control of seizures.

Decanoic acid is a major constituent of the MCT ketogenic diet, providing ∼40% of the medium chain fat within the diet (Sills *et al.*, 1986*a*). Even though decanoic acid is extensively metabolized to carbon dioxide, ketone bodies, and long chain fatty acids, it has been reported that there are high concentrations of decanoic acid (averaging 156.7 µM) in the serum of children with intractable epilepsy treated with MCT diet for the control of drug-resistant seizures (Haidukewych *et al.*, 1982; Sills *et al.*, 1986*a*; Dean *et al.*, 1989). A direct correlation between decanoic acid concentrations and seizure control has not, however, been shown, partly because the studies are too small to demonstrate such a correlation, but also because the ketogenic diet may have multiple other actions. Importantly, mice treated with decanoic acid by gastric gavage (30 mmol/kg; Wlaz *et al.*, 2012) have increased brain decanoic acid concentrations (up to 240 µM), which represent 60–80% of serum levels. These results indicate that fatty acids are present in appreciable amounts in the peripheral blood and brain and therefore are ideally placed to have an effect on seizure control in the brain. Decanoic acid has also been shown to delay the onset of picrotoxin-induced clonic seizures and prolong the survival time in mice with pentylenetetrazol-induced convulsions (Nakamura *et al.*, 1990). In addition, there is a direct effect of the fatty acids contained in MCT on cerebral excitability (Huttenlocher *et al.*, 1971; Sills *et al.*, 1986*a*, *b*). As we also show that decanoic acid inhibits epileptiform activity *in vitro*, it is likely that decanoic acid is a significant therapeutic component of the diet.

We determined the acute effect of decanoic acid on synaptic transmission in the CA1 area of the rat hippocampus and identified that decanoic acid reduced EPSC amplitude, at a similar concentration to peak plasma concentrations in children undergoing the diet (300 µM; Haidukewych *et al.*, 1982; Sills *et al.*, 1986*a*; Dean *et al.*, 1989) and in a manner consistent with a postsynaptic effect. Moreover, the same concentration of decanoic acid had no appreciable effect on inhibitory transmission. These data indicate that decanoic acid acts at AMPA receptors. These receptors provide a recognized target for seizure control, and mediate fast glutamatergic synaptic transmission in the CNS (Traynelis *et al.*, 2010; Rogawski, 2011). AMPA receptors play a key role in generating and propagating epileptic activity and, in the long-term, adaptive cellular plasticity associated with epileptogenesis (Rogawski and Donevan, 1999; Chapman, 2000). The receptors are present in all areas of the brain relevant to epilepsy, including the cerebral cortex, amygdala, thalamus and hippocampus (Beneyto and Meador-Woodruff, 2004; Rogawski, 2011). Furthermore, AMPA receptor antagonists have a broad spectrum of anticonvulsant activity in various *in vitro* and *in vivo* epilepsy models (Rogawski and Donevan, 1999; Rogawski, 2011). Here we show that the degree of AMPA receptor antagonism by decanoic acid is sufficient to explain its antiseizure effect. Similarly, perampanel, a recently approved treatment for refractory partial epilepsy, acts through AMPA receptor antagonism (Rektor, 2013). Thus, our results showing that decanoic acid reduces the magnitude of fast glutamatergic signalling in the hippocampus are consistent with previous reports that AMPA receptors are a recognized target for antiseizure therapies (Meldrum and Rogawski, 2007; Szenasi *et al.*, 2008; Russo *et al.*, 2012).

The results from *ex vivo* hippocampal slices do not, however, distinguish a direct from an indirect effect of decanoic acid on AMPA receptors. To overcome this, we used a heterologous expression model, where AMPA receptors were expressed in *Xenopus* oocytes to enable a detailed characterization of the effect of decanoic acid on AMPA receptor activity. Decanoic acid directly inhibited AMPA receptors comprised of GluA1 homodimers, and GluA1/2 and GluA2/3 heterodimers—the latter two represent the two most abundant AMPA receptor combinations in the adult brain (Santos *et al.*, 2009; Wang *et al.*, 2012). It is interesting to note that patients with chronic epilepsy show a significant increase of hippocampal dendritic GluA2/3 subunits in dentate granule cells (de Lanerolle *et al.*, 1989), and our data would suggest a concomitant enhanced efficacy of decanoic acid in this population. GluA subunits show differential spatial and temporal changes in subunit expression throughout development (Talos *et al.*, 2006), and the GluA2/3 subunits are expressed at increased levels during postnatal development (postnatal Days 10–15) in the rat hippocampus (Arai *et al.*, 1997), and long-term expression changes following the development of epilepsy in animal models (Friedman *et al.*, 1994). These results indicate that decanoic acid exerts a direct effect on AMPA-mediated currents, at concentrations as low as 100 µM (providing ∼20% inhibition), suggesting a likely influence on neuronal function in patients on the MCT ketogenic diet. However, the finding that decanoic acid likely has additional therapeutic targets due to its effect on phosphoinositides (Chang *et al.*, 2012, 2014*a*, *b*), raises the question of whether this degree of AMPA receptor antagonism is sufficient to explain its therapeutic effects in humans. Perampanel, a selective AMPA receptor antagonist, at the free concentrations observed to be efficacious in humans, 30–50 nM (Rogawski and Hanada, 2013), results in ∼20% reduction in field EPSP slope (Ceolin *et al.*, 2012). This effect is of a similar magnitude to the effect that we observed with therapeutically relevant concentrations of decanoic acid, indicating that the AMPA receptor antagonism may be sufficient to explain the antiseizure effects in humans (although we cannot exclude that its' effect on other targets could also play a part).

We continued our investigation into AMPA receptor inhibition by further characterizing the mechanism of decanoic acid inhibition, in particular determining whether it competes with glutamate for binding to the AMPA receptor. This is relevant as during conditions in which there are large rises in glutamate concentrations (such as seizure activity), competitive inhibitors may be less effective. However, we clearly demonstrate that varying the glutamate concentration by an order of magnitude does not alter the IC50 of decanoic acid inhibition, indicating that decanoic acid is a non-competitive inhibitor at AMPA receptors. The potency of decanoic acid-dependent AMPA receptor inhibition is therefore independent of increased glutamate during seizure activity and will still occur at synaptic glutamate concentrations in the millimolar range (Clements *et al.*, 1992). We also determined that AMPA receptor inhibition by decanoic acid is voltage-dependent, and is more effective at depolarized potentials. This voltage dependence suggests that the therapeutic effect of decanoic acid in reducing AMPA receptor currents is enhanced during post-synaptic activation and seizure propagation. This result also suggests that the binding site for decanoic acid lies in or near to the channel region of the receptor and is consistent with the properties of known AMPA receptor pore blockers such as the polyamines (Washburn and Dingledine, 1996).

Our modelling of the binding of decanoic acid to AMPA receptors also suggests a binding site in the channel region of the receptor. Our data indicate that decanoic acid binds by means of hydrogen bonding between its two carboxylic acid oxygens and the two hydrogens of the cyclic amine group of proline at residue position 584 on each of the four M3 helices subunits within the ion channel. It is likely that the binding of decanoic acid to each of the four subunits cooperatively contributes to its therapeutic effects. We believe that this mode of action will provide both steric effects restricting the physical dimensions of the ion channel pore coupled to electrostatic interactions between the negatively charged carboxylic group and cations flowing through the ion channel. Interactions between decanoic acid and cations passing through the channel would not only result in further narrowing the ion channel pore, but also create a positive electrostatic field which may serve to repel and prevent additional cations from passing through the blocked ion channel. However, the precise mechanistic aspects of decanoic acid function, including possible effect on receptor desensitization, remain unclear. The distinct localization of binding for decanoic acid and perampanel (Szenasi *et al.*, 2008; Rogawski, 2011) suggests different inhibitory profiles and, possibly, clinical effects. This may explain why certain adverse effects of perampanel, such as increased aggression (Rugg-Gunn, 2014; Steinhoff *et al.*, 2014), are not so evident with the MCT ketogenic diet. Moreover, as perampanel and decanoic acid act at separate sites, it is possible that they have a cooperative effect at the AMPA receptor, suggesting that permapanel and the ketogenic diet could be synergistic.

Our results have far-reaching clinical implications. For Lennox-Gastaut syndrome, Doose syndrome and Dravet’s syndrome antiepileptic drugs are often insufficient to obtain seizure control and non-pharmacological interventions are often required. Here the MCT diet can be effective in seizure management (Kossoff and Rho, 2009; Vanstraten and Ng, 2012; Laux and Blackford, 2013), and enhanced dietary intake of decanoic acid may provide additional therapeutic benefit. Furthermore, treatment of adults with drug-resistant epilepsy, who show poor compliance to the stringent dietary regimen necessary for the MCT diet, may better tolerate a normal diet with just added decanoic acid, e.g. in the form of a triglyceride.

There are a range of limitations to the discovery of an acute effect of decanoic acid on seizure control. First, it is likely that decanoic acid also has chronic effects, as has been shown recently on mitochondrial proliferation (Hughes *et al.*, 2014), and these effects may also have roles in decanoic acid’s therapeutic function. In addition, the role of metabolism on elevated blood levels of decanoic acid (Rho and Sankar, 2008; Rho and Stafstrom, 2011) remains unclear. Is a reduction in carbohydrate load necessary for elevated blood decanoic acid levels? Recent studies have suggested that dietary MCT oil, concomitant with a non-restricted carbohydrate diet, gave rise to ketosis (Courchesne-Loyer *et al.*, 2013), although changes in specific medium chain fatty acids in the plasma were not determined. Future studies monitoring decanoic acid in the plasma of patients or healthy individuals on different composition MCT oils, or with differing carbohydrate intakes, should provide indications of the dietary conditions necessary to elevate plasma decanoic acid levels to provide seizure protection through direct AMPA receptor inhibition.

The direct effect of decanoic acid on AMPA receptor mediated currents raises a concern of a detrimental effect on cognitive function. Numerous studies have demonstrated that AMPA receptors contribute to synaptic strengthening during long-term potentiation, a cellular model of synaptic plasticity, and experience-dependent neuronal plasticity (Talos *et al.*, 2006; Santos *et al.*, 2009; Wang *et al.*, 2012). This is further supported by mouse models, lacking the gene encoding the GluA1 subunit (*Gria1*) exhibiting impaired hippocampus-dependent memory (Sanderson *et al.*, 2008). However, neither competitive nor non-competitive antagonists of AMPA receptors (at concentrations that inhibit seizure activity) had an effect on long-term potentiation (a cellular correlate of learning and memory) (Sanderson *et al.*, 2008), and this is consistent with *in vivo* data in which AMPA receptor antagonists at therapeutic doses do not affect cognition (Pan *et al.*, 2010). Indeed, in humans, the MCT ketogenic diet has been shown to have diverse positive effects on brain function, such as increased alertness, better cognitive functioning, and improved behaviour, not only in epilepsy patients (Kinsman *et al.*, 1992; Pulsifer *et al.*, 2001) but also in patients with type 1 diabetes given an insulin infusion (Page *et al.*, 2009). The extent to which these effects can be attributed to decanoic acid or other components of the diet remains to be determined.

## Funding

We gratefully acknowledge an NC3Rs grant G0900775 to R.S.B.W. and M.W. to support this research, and PhD studentship to K.A. by Vitaflo Ltd. P.E.C. is grateful for funding from the Royal Society. Part of this work was undertaken at UCLH/UCL which receives a proportion of funding from the Department of Health’s NIHR Biomedical Research Centers funding scheme.

## Supplementary material

Supplementary material is available at *Brain* online.

## Supplementary Material

Supplementary Data

## Abbreviations

AMPA: α-amino-3-hydroxy-5-methyl-4-isoxazolepropionic acid
EPSC: excitatory postsynaptic current
Glu: ionotropic glutamate (AMPA) receptors
IPSC: inhibitory postsynaptic current
MCT: medium chain triglyceride
NMDA: *N*-methyl D-aspartic acid
