# Supplementary material for: Seizure control by decanoic acid through direct AMPA receptor inhibition
Source: Brain. 2015 Nov 25;139(2):431–43. doi: 10.1093/brain/awv325 (PMC4805082; doi:10.1093/brain/awv325)
Supplement: Supplementary Data [file awv325_supplementary_data.zip › UPDATED_SUPPLEMENTARY_FIGURE.pdf]

Supplementary Figures

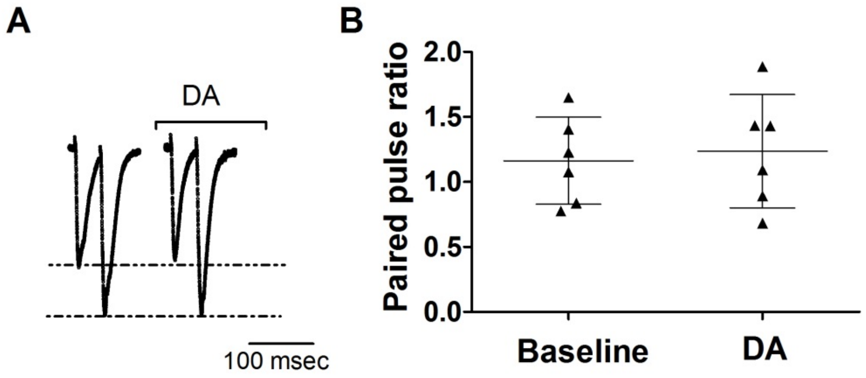

Figure S1: Decanoic acid does not alter the paired pulse ratio. Currents were recorded from hippocampal CA1 pyramidal cells following exposure to decanoic acid (DA; 300 μM). **(A)** Scaled representative traces of the paired pulse ratio before and after application of decanoic acid, and **(B)** paired pulse ratio from 6 cells, where no significant change in paired pulse ratio was found ($p = 0.69$, $n = 6$, students t-test), supporting a post-synaptic site of action for decanoic acid. Graphs show means ± SD.

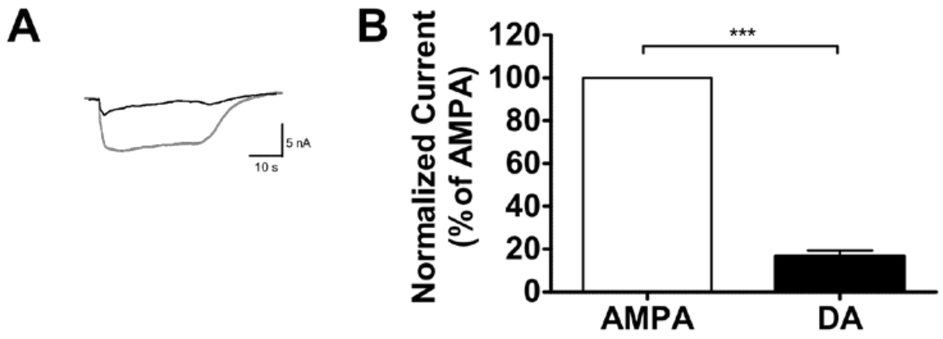

Figure S2: Decanoic acid inhibits AMPA (GluA2/GluA3) mediated current evoked by the selective agonist AMPA. (A) Representative traces from electrophysiological recordings showing the effect of decanoic acid (1 mM) on trace of GluA2/GluA3 mediated current induced by AMPA (30µM). Traces for AMPA evoked inward currents in the absence of decanoic acid (grey) and presence of decanoic acid (black). Oocytes were voltage-clamped at a holding potential of −50 mV. **(B)** Comparison of the mean of AMPA-induced current from oocytes expressing GluA2/GluA3 receptors in absence and presence of decanoic acid. Graphs show means ± SEM. Statistical analysis was performed using paired student-t test. *** Indicates a significant difference at $P<0.001$.

A

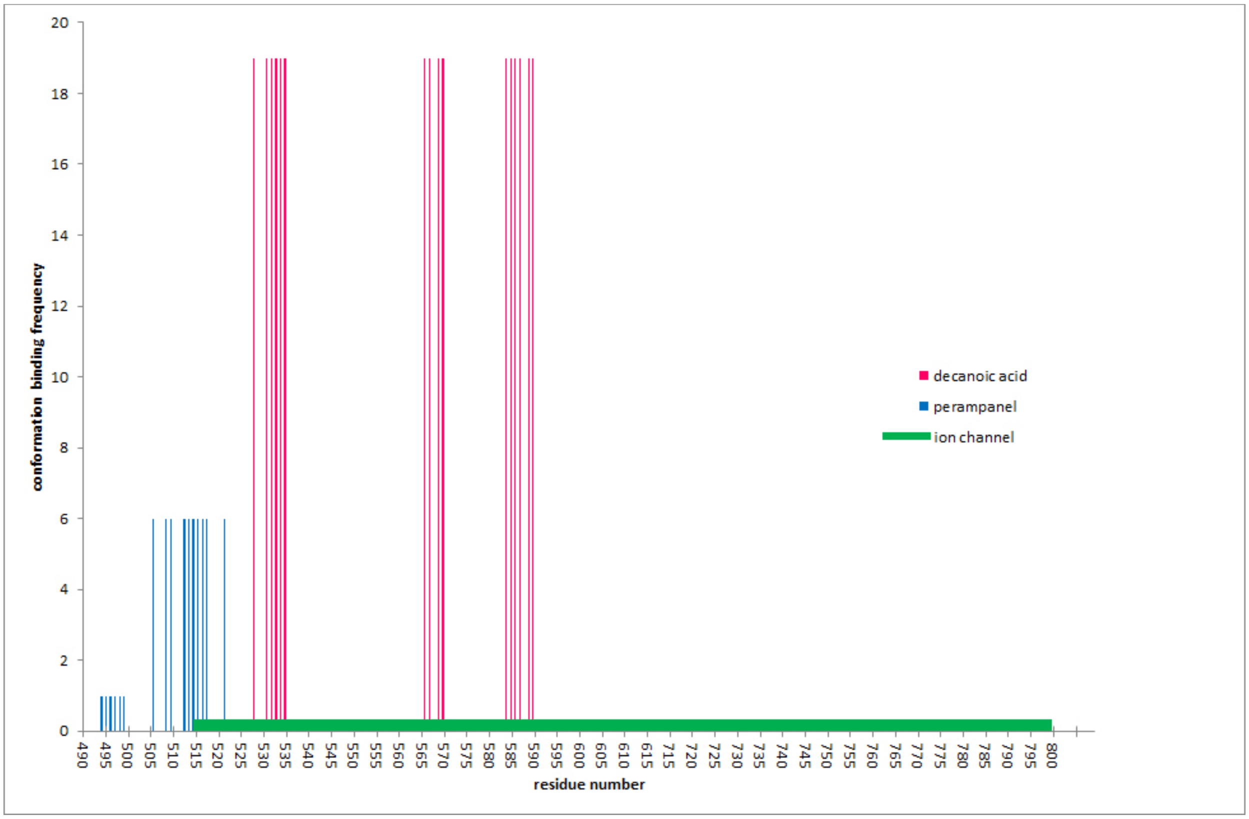

B

NSIQIGGLFPRGADQEYSAFRVGMVQFSTSEFRLTPHIDNLEVANSFAVTNAFCSQFSRGVYAIFGFYDK
KSVNTITSFCGTLHVSFITPSFPTDGTHPFVIQMRPDLKGALLSLIEYYQWDKFAYLYDSDRGLSTLQAV
LDSAAEKKWQVTAINVGNINNDKKDETYRSLFQDLELKKERRVILDCERDKVNDIVDQVITIGKHVKGYH
YIIANLGFTDGDLLKIQFGGAEVSGFQIVDYDDSLVSKFIERWSTLEEKEYPGAHTATIKYTSALTYDAV
QVMTEAFRNLRKQRIEISRRGNAGDCLANPAVPWGQGVEIERALKQVQVEGLSGNIKFDQNGKRINYTIN
IMELKTNGPRKIGYWSEVDKMVLTEDDTSGLEQKTVVVTTILESPYVMMKANHAALAGNERYEGYCVDLA
AEIAKHCGFKYKLTIVGDGKYGARDADTKIWNGMVGELVYGKADIAIAPLTITLVREEVIDFSKPFMSLG
ISIMIKKPQKSKPGVFSFLDPLAYEIWMCIVFAYIGVSVVLFLVSRFSPYEWHTEEFEDGRETQSSESTN
EFGIFNSLWFSLGAFMQQGADISPRSLSGRIVGGVWWFFTLIIISSYTANLAAFLTVERMVSPIESAEDL
SKQTEIAYGTLDSGSTKEFFRRSKIAVFDKMWTYMRSAEPSVFVRTTAEGVARVRKSKGKYAYLLESTMN
EYIEQRKPCDTMKVGGNLDSKGYGIATPKGSSLGTPVNLAVLKLSEQGLLDKLKNKWWYDKGECGAKDSG
SKEKTSALSLSNVAGVFYILVGGLGLAMLVALIEFCYKSRAEAKRMKGLVPRG

Figure S3: Modeling the binding of decanoic acid to AMPA receptors. (A) Participation frequency of amino acid residues within 6 Angstroms of each of the ligands bound to 3KG2. Bars indicate the number of different conformations binding at the given residues for the color-coded ligand for decanoic acid (magenta) and perampanel (blue). The green line demarks those residues making up the ion channel in the transmembrane domain of 3KG2 to facilitate identifying potential ligand binding sites found specifically within the ion channel. (B) Primary sequence of 3KG2, with the highlighted residues as proposed binding site for decanoic acid (magenta), perampanel (blue), found to be within 6 angstroms of the docked ligands. The residues highlighted in yellow demark the binding site of perampanel as reported in the literature (Szenasi *et al.*, 2008).
