# Supplementary figures and images for: Seizure control by decanoic acid through direct AMPA receptor inhibition
Source: Brain. 2015 Nov 25;139(2):431–43. doi: 10.1093/brain/awv325 (PMC4805082; doi:10.1093/brain/awv325)

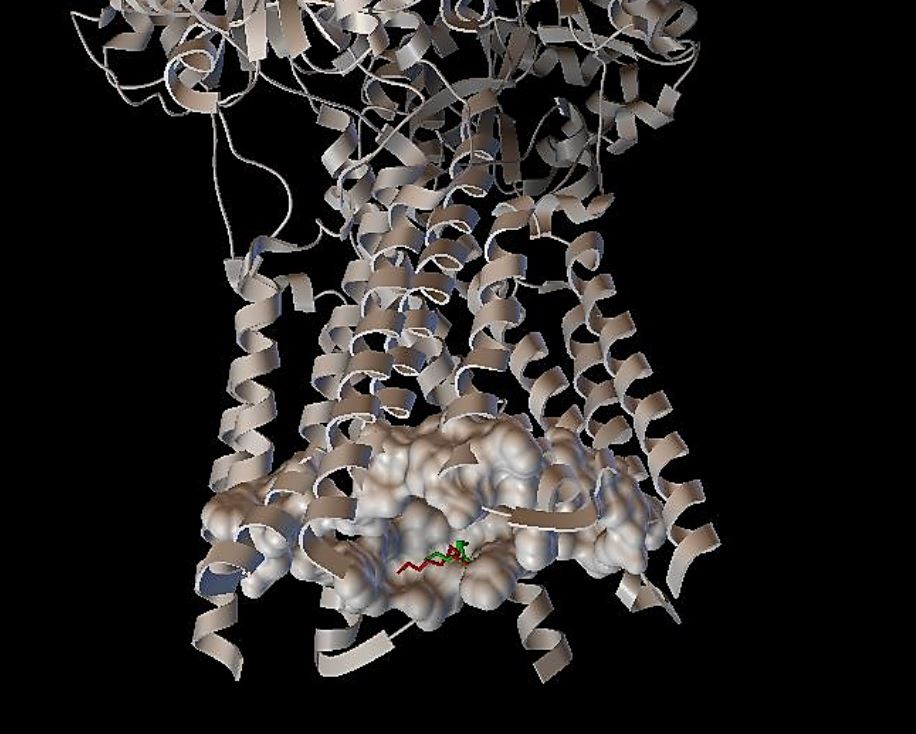

Supplement: Supplementary Data [file awv325_supplementary_data.zip › brain-2015-01396-File010.JPG]
